# The SARS‐CoV‐2 main protease (M^pro^): Structure, function, and emerging therapies for COVID‐19

**DOI:** 10.1002/mco2.151

**Published:** 2022-07-14

**Authors:** Qing Hu, Yuan Xiong, Guang‐Hao Zhu, Ya‐Ni Zhang, Yi‐Wen Zhang, Ping Huang, Guang‐Bo Ge

**Affiliations:** ^1^ Shanghai Frontiers Science Center of TCM Chemical Biology Institute of Interdisciplinary Integrative Medicine Research Shanghai University of Traditional Chinese Medicine Shanghai China; ^2^ Clinical Pharmacy Center Cancer Center Department of Pharmacy Zhejiang Provincial People's Hospital Affiliated People's Hospital Hangzhou Medical College, Hangzhou Zhejiang China

**Keywords:** 3‐chymotrypsin‐like protease (3CL^pro^), broad‐spectrum anti‐coronavirus agents, SARS‐CoV‐2, β‐coronavirus 3CL^pro^ inhibitor

## Abstract

The main proteases (M^pro^), also termed 3‐chymotrypsin‐like proteases (3CL^pro^), are a class of highly conserved cysteine hydrolases in β‐coronaviruses. Increasing evidence has demonstrated that 3CL^pro^s play an indispensable role in viral replication and have been recognized as key targets for preventing and treating coronavirus‐caused infectious diseases, including COVID‐19. This review is focused on the structural features and biological function of the severe acute respiratory syndrome coronavirus 2 (SARS‐CoV‐2) main protease M^pro^ (also known as 3CL^pro^), as well as recent advances in discovering and developing SARS‐CoV‐2 3CL^pro^ inhibitors. To better understand the characteristics of SARS‐CoV‐2 3CL^pro^ inhibitors, the inhibition activities, inhibitory mechanisms, and key structural features of various 3CL^pro^ inhibitors (including marketed drugs, peptidomimetic, and non‐peptidomimetic synthetic compounds, as well as natural compounds and their derivatives) are summarized comprehensively. Meanwhile, the challenges in this field are highlighted, while future directions for designing and developing efficacious 3CL^pro^ inhibitors as novel anti‐coronavirus therapies are also proposed. Collectively, all information and knowledge presented here are very helpful for understanding the structural features and inhibitory mechanisms of SARS‐CoV‐2 3CL^pro^ inhibitors, which offers new insights or inspiration to medicinal chemists for designing and developing more efficacious 3CL^pro^ inhibitors as novel anti‐coronavirus agents.

## Graphical abstract

A comprehensive summary of recent advances in SARS‐CoV‐2 3CL^pro^ inhibitors (including marketed drugs, peptidomimetic, and non‐peptidomimetic synthetic compounds, as well as natural compounds and their derivatives), including the inhibitory activities, inhibitory mechanisms, and key structural features, provides new insights for designing and developing more efficacious 3CL^pro^ inhibitors as broad‐spectrum anti‐coronavirus agents.

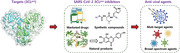

## 1 INTRODUCTION

Coronaviruses (CoVs) are single‐stranded positive‐sense ribonucleic acid (RNA) enveloped viruses with a 5′‐cap and 3′‐poly‐A tail that can be classified into four subgroups: α, β, 𝛾, and δ. The hosts of CoVs are vertebrates that range from human beings to birds, generally causing respiratory and gastrointestinal tract disorders.^1^, ^2^, ^3^, ^4^, ^5^, ^6^ Seven human coronaviruses have emerged, including three fatal β‐CoVs (severe acute respiratory syndrome coronavirus [SARS‐CoV], Middle‐East respiratory syndrome coronavirus [MERS‐CoV], and severe acute respiratory syndrome coronavirus 2 [SARS‐CoV‐2]).^7^, ^8^, ^9^, ^10^ Among them, SARS‐CoV‐2 and SARS‐CoV belong to the subgenus *Sarbecovirus* of β‐CoVs according to the latest release of the International Committee on Taxonomy of Viruses (https://talk.ictvonline.org/). Particularly, SARS‐CoV‐2, the pathogen for coronavirus disease 2019 (COVID‐19), has taken millions of lives, generating a huge negative impact on the public.^11^, ^12^, ^13^, ^14^ To combat this epidemic effectively, scientists have made great efforts in drug repurposing, vaccine development, and novel medication discovery. To date, several effective vaccines that mainly target the viral spike (S) protein can be used for the preliminary prevention of COVID‐19 by eliciting an immune response.^15^, ^16^ Newly emerging variants (e.g., Delta and Omicron) of SARS‐CoV‐2 have generated high‐frequency mutations in the S protein, including nucleic acid mutations and amino acid mutations, which present potential hazards for the effectiveness of vaccines and mutation‐mediated resistance.^17^, ^18^, ^19^, ^20^

**FIGURE 1:**
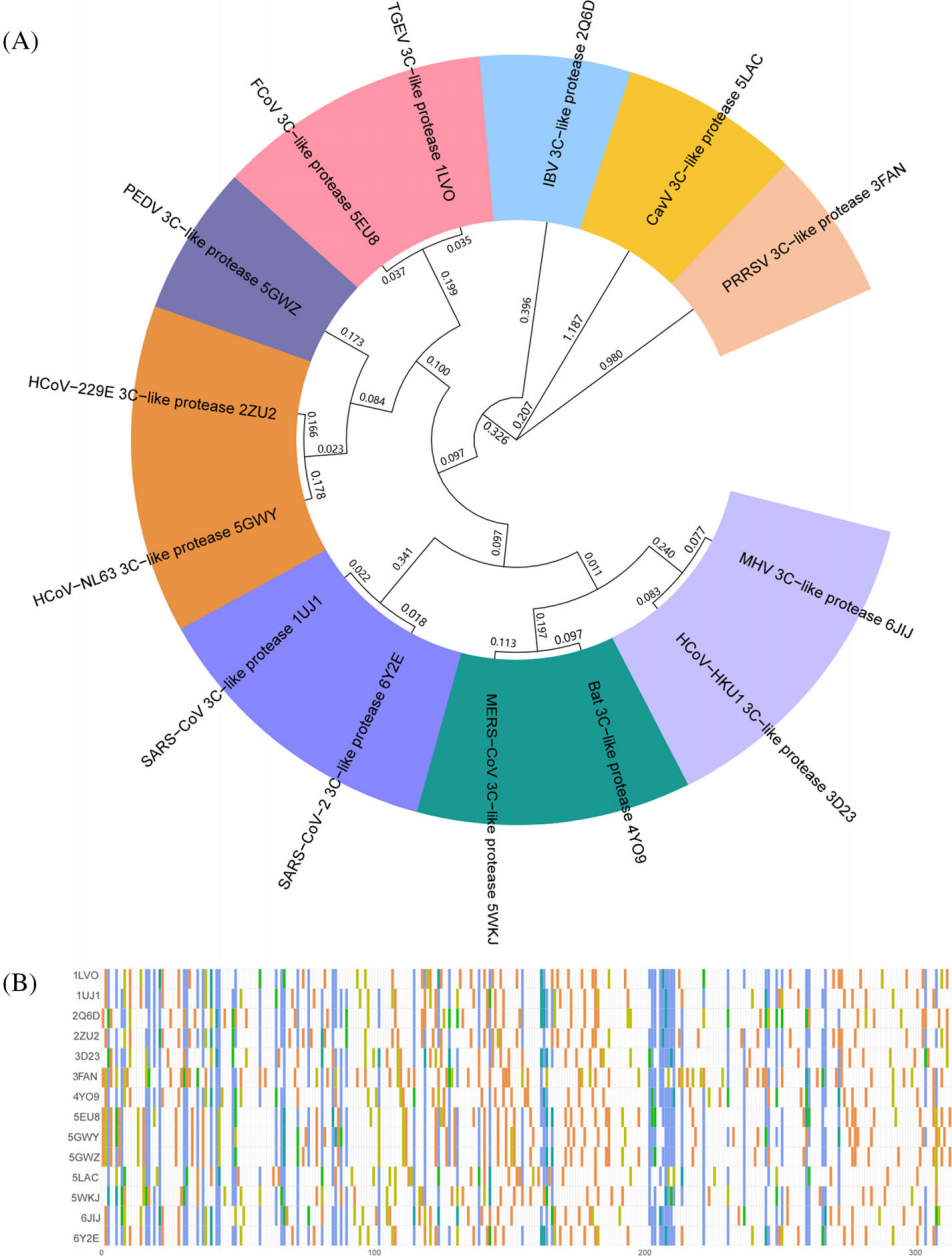
Phylogenetic relationships for 14 reported 3‐chymotrypsin‐like proteases (3CL^pro^s) in *Nidovirus*. (A) The evolutionary distances (genetic variations) of 3CL^pro^s are presented on branches. (B) Amino acid homologous sequence alignment of 3CL^pro^s

In the process of virus multiplication, the main proteases (M^pro^, also known as 3CL^pro^), a class of highly conserved cysteine hydrolases from CoVs, are capable of cleaving polyproteins at multiple sites to yield multiple functional proteins.^21^ Considering that 3CL^pro^s play a vital role in CoV replication, especially in the two of the most serious pandemics of the 21st century caused by SARS‐CoV‐2 and SARS‐CoV, these key hydrolases have been validated as promising targets for developing broad‐spectrum anti‐CoV agents.^22^, ^23^, ^24^, ^25^, ^26^ Because no homolog of 3CL^pro^ has been identified in humans, it is feasible to develop efficacious and specific 3CL^pro^ inhibitors with extremely weak inhibitory effects on human proteases, thereby reducing the side effects caused by 3CL^pro^ inhibitors. As shown in Figure 1, the phylogenetic relationships of 14 kinds of 3CL^pro^s from coronaviruses show that the relatedness of the 3CL^pro^s for SARS‐CoV‐2 and SARS‐CoV are extremely close^27^ ^–^ ^37^; thus, most attempts to develop new SARS‐CoV‐2 3CL^pro^ inhibitors are based on previously reported SARS‐CoV 3CL^pro^ inhibitor. As an attractive target for combating viral replication and pathogenesis to control various CoVs, 3CL^pro^ has drawn much interest from both academics and industry.

In recent years, multiple drug discovery strategies have been utilized to find or develop a number of 3CL^pro^ inhibitors against SARS‐CoV‐2, such as drug repurposing, virtual screening coupled with high‐throughput screening (HTS), and structure‐based drug design.^38^, ^39^, ^40^ Moreover, the discovery of active compounds from natural products remains one of the most important sources for developing novel anti‐CoV agents.^41^, ^42^, ^43^, ^44^, ^45^, ^46^, ^47^ Therefore, many research groups have devoted their efforts to finding anti‐CoV agents in naturally occurring compounds.^48^, ^49^, ^50^, ^51^, ^52^ To date, a variety of marketed drugs and other structurally diverse synthetic compounds, as well as a number of natural compounds, have been found to be efficacious inhibitors of SARS‐CoV‐2 3CL^pro^, showing great potential for developing novel broad‐spectrum anti‐CoV agents.^53^, ^54^, ^55^, ^56^, ^57^, ^58^, ^59^ Thus, this review focuses on the structural features and function of 3CL^pro^ and recent advances in the discovery of SARS‐CoV‐2 3CL^pro^ inhibitors, aiming to provide a SARS‐CoV‐2 3CL^pro^ inhibitor library for medicinal chemists to design and develop more efficacious anti‐CoV agents in the future.

## 2 STRUCTURAL FEATURES AND FUNCTION OF 3CL^pro^

A total of 432 structures of SARS‐CoV‐2 3CL^pro^ are currently uploaded to the PDB database, containing 54 apoprotein structures and 378 liganded protein structures (Supporting Information). The available structures of SARS‐CoV‐2 3CL^pro^ were crystallized at temperatures ranging from 277 to 300 K and refined at resolutions ranging from 1.2 to 2.98 Å. 3CL^pro^ is approximately 34.21 kDa per monomer (average molecular weight of monomeric deposited models). 3CL^pro^ is matured in a dimeric form, and the individual monomers are enzymatically less active, where the monomers consist of three domains, including domain I, domain II, and domain III.^60^, ^61^ Among them, domain III is an extra helix domain, whose aggregation initiates the dimerization of 3CL^pro^.^16^, ^22^, ^36^, ^62^ Generally, the monomer of 3CL^pro^ is a transient state that proved to be enzymatically less active, while the dimeric form acts as a functional unit with the highest hydrolytic activity (Table 1).^63^, ^64^, ^65^ The firm binding between the N‐finger and C‐terminus is one of the key conditions for the formation of dimeric 3CL^pro^, especially the salt bridge between Arg4 and Arg298.^66^, ^67^, ^68^, ^69^, ^70^, ^71^, ^72^

**TABLE 1:** Molecular features of 3‐chymotrypsin‐like proteases (3CL^pro^) from severe acute respiratory syndrome coronavirus 2 (SARS‐CoV‐2) and severe acute respiratory syndrome coronavirus (SARS‐CoV)

Property	SARS‐CoV‐2 3CL^pro^	SARS‐CoV 3CL^pro^
Molecular weight (kDa)	34	34
Isoelectric point	6.0	6.2
Optimal pH	7.5	7.0
Length of monomer (residue)	306	306
Mature form	Homodimer	Homodimer
Catalytic residues	His41, Cys145	His41, Cys145

**FIGURE 2:**
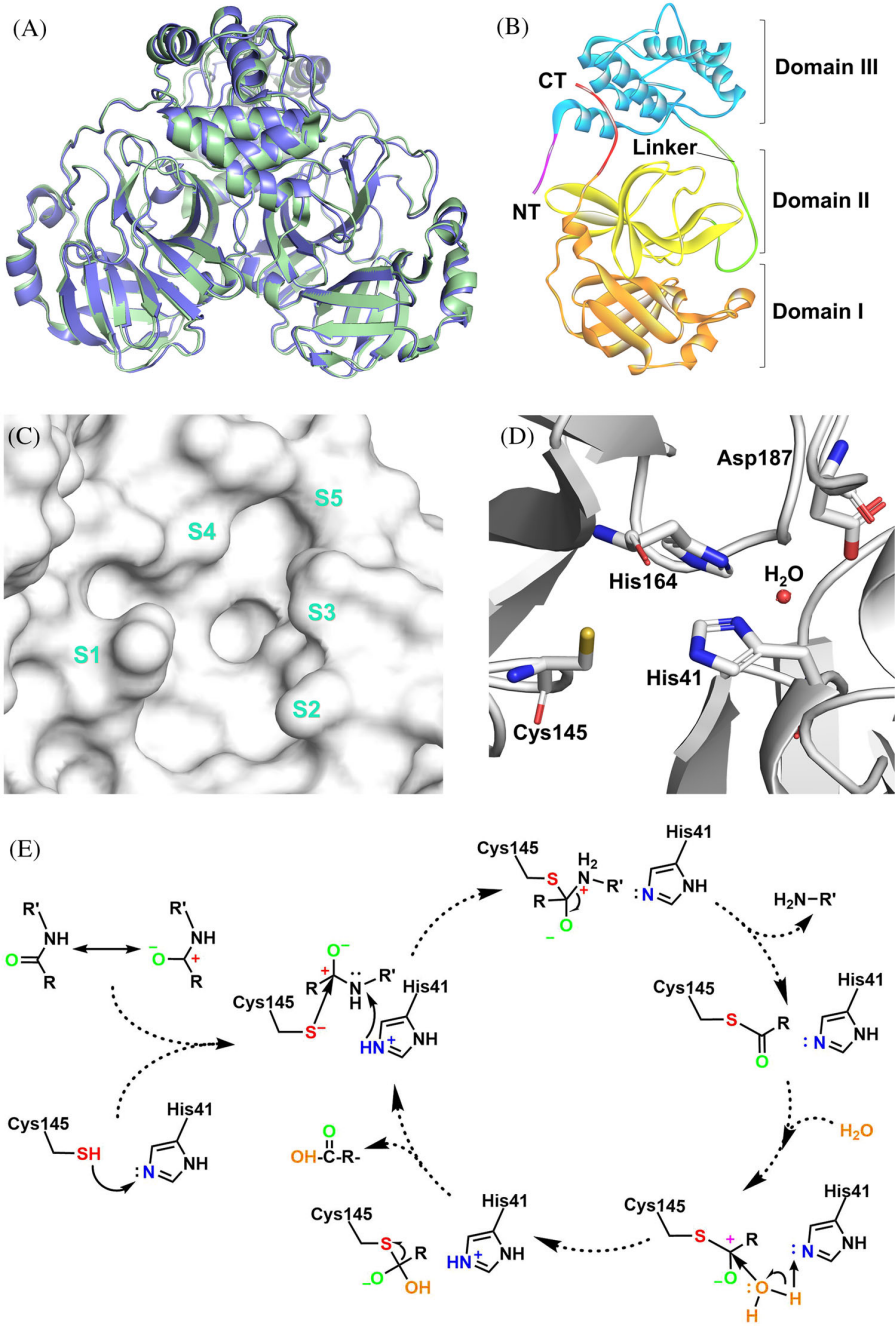
(A) The 3D structure of severe acute respiratory syndrome coronavirus 2 (SARS‐CoV‐2) 3CL^pro^ (pale green, PDB: 6XHU) and severe acute respiratory syndrome coronavirus (SARS‐CoV) 3CL^pro^ (slate, PDB: 1UJ1). (B) Three structural domains (domain I: orange, domain II: yellow, domain III: blue) of SARS‐CoV‐2 3CL^pro^ monomer. (C) The surface representation for the catalytic pocket (sub‐pockets: S1–S5) of SARS‐CoV‐2 3CL^pro^. (D) The amino acid residues in the active site of SARS‐CoV‐2 3CL^pro^. (E) The catalytic mechanism of 3CL^pro^ on the hydrolysis of amide substrate

The catalytic site of 3CL^pro^ is located at the intersection of domains I and II, which can be divided into mainly five sub‐pockets, including S1, S2, S3, S4, and S5 (Figure 2).^36^, ^73^ The key facial residues of five sub‐pockets are listed in Table S1, whose dimensional chemical environment matches five specific substrate‐binding positions.^64^, ^74^, ^75^ P1, P2, and P1ʹ positions mainly determine the substrate specificity of 3CL^pro^, while P4, P3, and P3ʹ boost the recognition and stable binding of substrates.^64^, ^76^ The O^β^ atom of glutamine could bind to the oxyanion hole (residues 143–145) of S1, and then the thiol of Cys145 could attack the C atom of glutamine as a nucleophile.^64^, ^77^ Therefore, P1 almost always requires glutamine or lactam warhead.^78^, ^79^, ^80^ Notably, only one of the catalytic sites possesses hydrolytic function in the homodimer.^16^, ^78^, ^81^

Different from the catalytic triad of 3‐chymotrypsin, the catalytic dyad of 3CL^pro^ is formed by Cys145 and His41.^82^, ^83^ The zwitter catalytic dyad Cys^–^145–His^+^41 needs to be activated by energetical water that is maintained by His164 and Asp187.^63^, ^84^, ^85^, ^86^, ^87^ Cleavage of the large polyprotein chains by 3CL^pro^ occurs at the glutamine residue in the P1 position of the substrate via a Cys145–His41 dyad, in which the cysteine thiol functions as the nucleophile in the proteolytic process. The cleavage of polyproteins by 3CL^pro^ using a universal nucleophilic‐type reaction mechanism is as follows (Figure 2E). Initially, the Cys145‐thiol on the catalytic dyad is deprotonated with the help of nearby His41, where the anionic sulfur attacks the C‐terminal C atom of the specially recognized Gln as a nucleophile.^88^ Then, after spitting the amide bond, the histidine restores the deprotonated form, and the generated thioester is attacked in an identical fashion, with water acting as the nucleophile leading to the release of the hydrolyzed C‐terminal, thus resetting the catalytic dyad.^89^, ^90^ The mutant experiment proved that the catalytic cysteine is essential to 3CL^pro^, as replacing cysteine with serine would result in a covalent product–enzyme complex or a covalent Ser145O^γ^–Gln306C bond, fatally blocking the self‐cleavage process.^64^

As a key cysteine protease, SARS‐CoV‐2 3CL^pro^ has 12 cysteine residues, but only three of them (Cys85, Cys145, and Cys156) are exposed to solvent.^91^ Catalytic Cys145 is the most important cysteine located in the catalytic site. Myricetin can bind to Cys145 in its oxidized form.^92^ Ebselen and its derivatives can modify Cys145 by forming a Se–S bond.^93^ Peptidomimetic α‐acyloxymethylketone warheads can react with Cys145 through a structure‐based selectivity mechanism.^94^ However, few covalent inhibitors have been discovered to be accessible to Cys85 and Cys156. Cys156 is only profiled using *N*‐ethylmaleimide, a small‐molecule electrophile that engages cysteine side‐chain thiolates by creating a covalent bond.^63^ The inconducive spatial environments enclosing Cys85 and Cys156 are speculated to be the cause. Although the bulk of the 3CL^pro^ cysteines is buried inside the protein, several cysteines are tested and predicted to be reactive. Cys22 and Cys44 are two conserved deprotonated cysteines in 3CL^pro^. Constant‐pH molecular dynamics (CpHMD) titration revealed that Cys22 and Cys44 are more nucleophilic than catalytic Cys145.^95^ Cys44 is largely inclined to be modified by flavonoids because the pocket encircled Cys44 is compatible with flavonoids, such as baicalein and covalent‐binding myricetin.^95^, ^96^ Cys300 is proven to be an allosteric site of 3CL^pro^.^95^ Myricetin and colloidal bismuth subcitrate (CBS) can bind to Cys300 of 3CL^pro^ and inhibit 3CL^pro^ as allosteric inhibitors.^96^, ^97^ Notably, a prominent dissociation of 3CL^pro^ occurs after incubation of CBS with 3CL^pro^, resulting in degradation of 3CL^pro^ and collapse of the active site.^97^

## 3 SYNTHETIC COMPOUNDS

With the help of the high‐resolution crystal structures of both SARS‐CoV‐2 3CL^pro^ and its homolog SARS‐CoV 3CL^pro^, a panel of computer‐aided drug design and crystallography‐guided fragment‐based drug discovery approach have been widely used to screen and design novel inhibitors against SARS‐CoV 3CL^pro^.^98^ A majority of synthetic SARS‐CoV‐2 3CL^pro^ inhibitors are designed based on the 3D structure of the active pocket and substrate preferences of the target enzyme, which could be structurally categorized into peptidomimetics and non‐peptidomimetics (small molecules).^40^, ^99^ Currently, a number of synthetic compounds (including peptidomimetics and non‐peptidomimetics) have been found with strong to extremely potent SARS‐CoV‐2 3CL^pro^ inhibitors, which have aroused significant interest in the pharmaceutical industry to develop more efficacious antiviral drug candidates with satisfying drug‐likeness properties and safety profiles for combating COVID‐19.^100^

### 3.1 Peptidomimetic SARS‐CoV‐2 3CL^pro^ inhibitors

**FIGURE 3:**
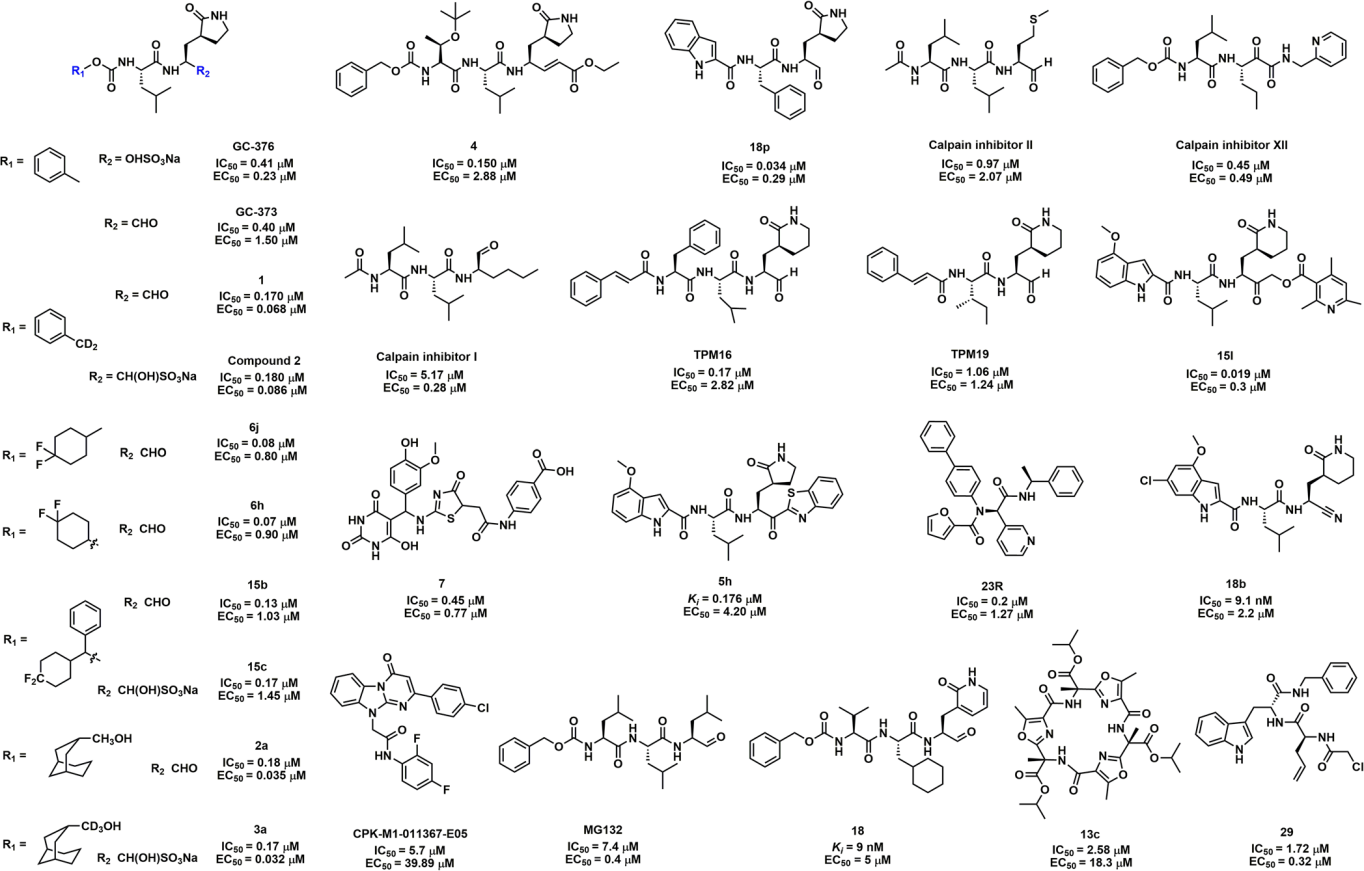
The chemical structures and half‐maximal inhibitory concentration (IC_50_) values for representative peptidomimetic SARS‐CoV‐2 3CL^pro^ inhibitors, as well as their half‐maximal effect concentration (EC_50_) values for anti‐SARS‐CoV‐2

Peptidomimetics have been widely used for the development of antiviral drugs, offering numerous properties, such as superior efficiency and safety, as well as less accumulation within the body.^101^ Thus, a series of peptidomimetic antiviral drugs have been rationally designed for the treatment of COVID‐19. As shown in Figure 3, GC376, a peptidomimetic antiviral protease inhibitor for the treatment of cats infected with feline infectious peritonitis virus, showed strong inhibition of SARS‐CoV‐2 3CL^pro^ and SARS‐CoV‐2 replication with the half‐maximal inhibitory concentration (IC_50_) of 26.4 nM and 0.91 μM, respectively.^102^, ^103^, ^104^ Vuong et al.^105^ also reported that this drug and its parent GC373 were potent inhibitors of 3CL^pro^ of SARS‐CoV and SARS‐CoV‐2 with IC_50_ values in the nanomolar range. Nuclear magnetic resonance (NMR) analysis showed that these inhibitors covalently modified Cys145 to reversibly form a hemithioacetal. Meanwhile, GC376 and compound **4** were found to be covalent inhibitors of SARS‐CoV‐2 3CL^pro^.^106^ Furthermore, Dampalla et al.^107^ synthesized a series of deuterated derivatives of GC376 and determined the therapeutic efficacy in a lethal mouse model. In the co‐crystal structure of SARS‐CoV 3CL^pro^, a novel stereocenter formed by compound **2** covalently attached to Cys145 with nearly the same hydrogen bonding interactions as SARS‐CoV‐2 3CL^pro^. As a result of the multiple advantages of introducing deuterium into the drug, the deuterated variants at the R‐site exhibited a significant increase in the anti‐3CL^pro^ and cell‐based assays, as well as improved pharmacokinetics and reduced toxicity.^107^, ^108^ Moreover, by using the fluorine‐walk approach to explore the binding modes of the F‐substituted phenyl ring, they found that compounds **15b** and **15c** were the most effective against SARS‐CoV‐2 3CL^pro^, with IC_50_ values of 0.13 and 0.17 μM, respectively.^109^ Compounds **6j** and **6h** inhibited SARS‐CoV‐2 3CL^pro^ with significant efficacy, while administration of compound **6j** significantly improved survival, reductions in lung virus titers, and lung histopathology throughout the day in a mouse model of MERS‐CoV infection.^80^ Several non‐deuterated and deuterated compounds containing a conformationally constrained cyclohexane moiety were synthesized, of which compound **2a/3a** displayed high potency in biochemical assays with IC_50_ values in the submicromolar range. Importantly, the half‐maximal effect concentration (EC_50_) values of compounds **2a** and **3a** against SARS‐CoV‐2 in Vero E6 cells were 0.035 and 0.032 μM, respectively.^110^ All of the above compounds contain GC376 variants with potent biological activity, making them potential anti‐COVID‐19 candidates.

A series of novel protease inhibitors with an aldehyde warhead targeting the 3C protease of enterovirus 71 was designed and synthesized, especially compound **18p**, which showed potent enzyme inhibitory activity and broad‐spectrum antiviral activity against a group of enteroviruses and rhinoviruses.^111^ Notably, compound **18p** showed strong replication inhibition against SARS‐CoV‐2 3CL^pro^ (IC_50_ = 0.034 μM, EC_50_ = 0.29 μM), making it a further potential candidate for the treatment of COVID‐19. In particular, calpain inhibitors I, II, and XII were identified as SARS‐CoV‐2 3CL^pro^ inhibitors in vitro and in vivo.^112^, ^113^ Moreover, five tetrapeptidomimetic anti‐3CL^pro^ inhibitors, similar to the backbone of **13a**, were successfully involved in the design of the catalytic dyad histidine residue (His41) of 3CL^pro^. Among them, TPM16 and TPM19 exhibited nanomolar inhibition and attenuated the cellular viral loads of SARS‐CoV‐2.^114^ Compound **15l** with novel α‐acyloxymethylketone warhead mimetic compounds was described by Bai et al.,^94^ which was identified to have potent SARS‐CoV‐2 3CL^pro^ and viral replication inhibition in vitro. Moreover, co‐crystallization of **15l** with SARS‐CoV‐2 3CL^pro^ confirmed the formation of covalent adducts. Compound **7** showed inhibition activity against 3CL^pro^, papain‐like protease (PL^pro^), and furin protease at IC_50_ values of 0.45, 0.085, and 0.29 μM, respectively. Moreover, compound **7** has a higher inhibitory effect on the virus and is nontoxic to mammalian cells, making it a powerful dual inhibitory activity against SARS‐CoV‐2.^115^ Using a covalent DNA‐encoded library screening platform, Ge et al.^116^ found that compound **1e** showed potent inhibition of SARS‐CoV‐2 3CL^pro^ (Table S2).

A small‐molecule compound **5h** containing an indole moiety was characterized against SARS‐CoV‐2 3CL^pro^ (inhibition constant, *K_i_* = 17.6 nM) via reversible covalent interactions. Based on Vero E6 cell assays, **5h** blocked the infectivity of SARS‐CoV‐2 with an EC_50_ value of 4.2 μM.^117^, ^118^ One novel SARS‐CoV‐2 3CL^pro^ inhibitor, compound **23R**, was highly selective compared to covalent inhibitors. The co‐crystal structure of SARS‐CoV‐2 3CL^pro^ with **23R** reveals a previously unexplored binding site located between the S2 and S4 pockets.^119^ Bai et al.^120^ described that compound **18b** bearing nitrile warheads displays good SARS‐CoV‐2 3CL^pro^ inhibition activity, which could reduce SARS‐CoV‐2 plaques in Vero E6 host cells (EC_50_ = 2.2 μM), and showed a better selectivity than the aldehyde warhead peptidomimetics for human cysteine proteases (cathepsins B, S, and L). Seven peptidomimetic SARS‐CoV‐2 3CL^pro^ inhibitors were identified from the Korea Chemical Bank library. Among these agents, CPK‐M1‐011367‐E05 showed strong anti‐3CL^pro^ activity and anti‐SARS‐CoV‐2 activity.^121^

For CoVs to successfully invade the host cell, the S protein of CoVs needs to be cleaved and activated by some host cell proteases, such as furin and transmembrane protease serine 2 (TMPRSS2). Cathepsin L is a lysosomal cysteine protease in the host that is closely related to the membrane fusion of SARS‐CoV.^122^ A clinical study suggested that cathepsin L level in COVID‐19 patients was positively correlated with disease course and severity.^123^ Specifically, the secondary cleavage of the S protein by cathepsin L promotes the cell–cell fusion of SARS‐CoV‐2, indicating that cathepsin L is a promising target for anti‐COVID‐19.^124^ Recently, a proteasome inhibitor, MG132, was identified as a dual inhibitor for SARS‐CoV‐2 3CL^pro^ and cathepsin L, which could covalently and reversibly bind to Cys145 of 3CL^pro^.^125^ A novel class of self‐masked aldehyde inhibitors for cruzain was developed, in which compound **18** showed extremely potent 3CL^pro^ inhibitory activity (*K_i_* = 9 nM) and good anti‐SARS‐CoV‐2 activity (EC_50_ = 5 μM) in A549/angiotensin‐converting enzyme 2 (ACE2) cells.^126^ Macrocyclic peptides are known for their higher membrane permeability, superior selectivity, and stability, making them a promising privileged structure in drug discovery. Macrocycle **13c** has been found to have significant inhibitory activity against SARS‐CoV‐2 3CL^pro^ (IC_50_ = 2.58 μM).^127^ Moreover, Johansen‐Leete et al.^128^ reported several high‐affinity thioether‐linked cyclic peptide inhibitors of SARS‐CoV‐2 3CL^pro^, and several inhibitors exhibited in vitro anti‐SARS‐CoV‐2 activity with EC_50_ values in the low micromolar range. Compound **29** was identified as a dual‐action inhibitor of SARS‐CoV‐2 proteases that inhibits 3CL^pro^ at a micromolar level (IC_50_ = 1.72 μM) while inhibiting PL^pro^ at a submicromolar level (IC_50_ = 0.67 μM).^129^

As shown in Table S2, a set of submicromolar covalent inhibitors with warheads were screened by Stille et al.,^130^ and compounds **16a** and **14a** significantly inhibited the catalytic activity of SARS‐CoV‐2 3CL^pro^. Breidenbach et al.^131^ identified and optimized two classes of protease inhibitors (azanitrile and pyridyl esters), of which azanitrile **8** (*K_i_* = 24 nM), equipped with a unique azanitrile warhead, was an irreversible inhibitor of SARS‐CoV‐2 3CL^pro^. SDZ224015, a promising clinical caspase‐1 inhibitor, was identified as a SARS‐CoV‐2 3CL^pro^ inhibitor (IC_50_ = 30 nM) and might form an irreversible covalent adduct with the target enzyme.^132^ MPI3 and MPI8 displayed high potency against SARS‐CoV‐2 3CL^pro^, with MPI8 showing the best selectivity toward host cathepsin L, reducing the potential toxicity toward host cells and high antiviral potency.^133^, ^134^ Using chlorofluoroacetamide as a reactive warhead, Yamane et al.^135^ have developed an irreversible inhibitor of SARS‐CoV‐2 3CL^pro^. Among them, the inhibitory activity of (*R*, *R*)‐18 against 3CL^pro^ was significantly higher than that of the other isomers.

### 3.2 Non‐peptidomimetic SARS‐CoV‐2 3CL^pro^ inhibitors

**FIGURE 4:**
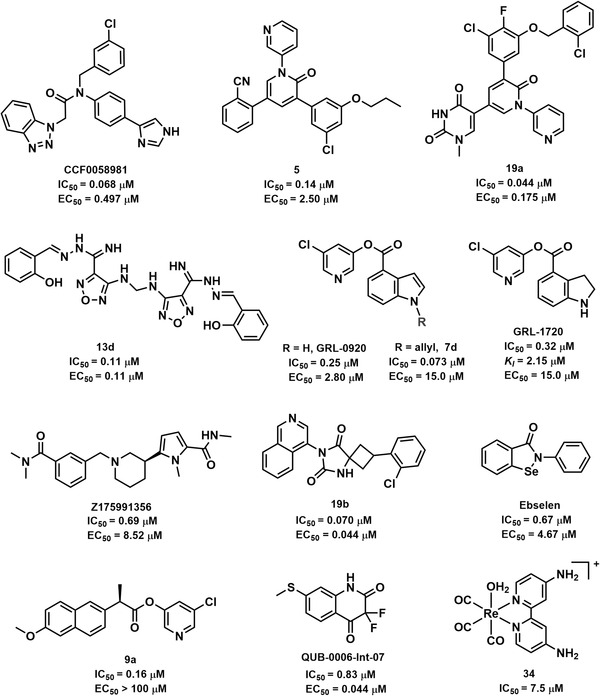
The structures and half‐maximal inhibitory concentration (IC_50_) values for representative non‐peptidomimetic SARS‐CoV‐2 3CL^pro^ inhibitors, as well as their half‐maximal effect concentration (EC_50_) values for anti‐SARS‐CoV‐2

To meet the urgent requirements for anti‐SARS‐CoV‐2 agents, scientists have made great efforts to discover anti‐SARS‐CoV‐2 agents from in‐house compound libraries or commercially available compounds via virtual screening coupled with experimental validation in early studies. The activities of non‐peptidomimetic SARS‐CoV‐2 3CL^pro^ inhibitors are summarized in Figure 4 and Table S2. For instance, Yang et al.^136^ adopted a multiple conformational‐based virtual screening strategy and surface plasmon resonance assay for SARS‐CoV‐2 3CL^pro^ inhibitors from a protein mimetics library. Six compounds presented inhibitory effects against 3CL^pro^ both in vitro and in HEK293T cells, and Z1759961356 hindered viral replication in Vero E6 cells with an EC_50_ value of 8.52 μM. Four isoquinolone‐based compounds from a database were reported as SARS‐CoV‐2 3CL^pro^ inhibitors with IC_50_ values of approximately 1 μM.^137^

In fact, some inhibitors have a potential impact on several 3CL^pro^s due to the high conservation of this protease among CoVs.^138^ For example, ML188 inhibited the 3CL^pro^ of SARS‐CoV, SARS‐CoV‐2, and porcine epidemic diarrhea virus, making it a promising broad‐spectrum antiviral agent.^74^ Moreover, structural optimization based on the reported inhibitor was a feasible strategy to develop SARS‐CoV‐2 3CL^pro^ inhibitors. CCF0058981, a novel compound derived from ML300 (SARS‐CoV 3CL^pro^ inhibitor),^139^ exerted a nanomolar level of activity (IC_50_ = 68 nM) against SARS‐CoV‐2 3CL^pro^, as well as superior anti‐SARS‐CoV‐2 activities in both cytopathic effect inhibition assays (EC_50_ = 0.497 μM) and plaque reduction assays (EC_50_ = 0.558 μM).^140^ Except for viral proteases, host proteases related to viral infection are also promising targets for fighting COVID‐19. In this context, Elseginy et al.^115^ focused on multi‐target inhibitors for fighting SARS‐CoV‐2. They demonstrated that not only compound **13d** inhibited 3CL^pro^ and PL^pro^ in SARS‐CoV‐2 but also furin protease in the host. Meanwhile, this compound could significantly inhibit SARS‐CoV‐2 (IC_50_ = 0.11 μM) in vitro.

Perampanel, an antiepileptic drug, showed weak inhibitory activity against SARS‐CoV‐2 3CL^pro^, while its cloverleaf motif could occupy three sub‐pockets with a high docking score, thus proposing it as a promising skeleton for novel 3CL^pro^ inhibitors.^141^ To validate this hypothesis, Zhang et al.^142^ put forward a useful strategy to guide the rational design of perampanel‐derived inhibitors, which was a combination of several methodologies, including the free‐energy perturbation calculation, structural analysis, biochemistry assessments, and X‐ray crystallography. Among the 27 analogs, compound **21b** exhibited a potent inhibitory effect against SARS‐CoV‐2 3CL^pro^ (IC_50_ = 18 nM) but with the greatest cytotoxicity. In particular, the combined use of compound **5** and remdesivir was initially predicted to have a synergistic effect on antiviral activity. To elevate the inhibitory activity and safety, they conducted crystallographic studies for further refinements.^143^ In a follow‐up study, this team found that 13 uracilyl‐containing compounds presented strong activity. Among 13 newly designed compounds, compound **19a** had potent enzyme inhibitory activities, good anti‐SARS‐CoV‐2 effects, good aqueous solubility, and low toxicity, suggesting that it is a promising compound for anti‐COVID‐19.^144^ According to the effective pharmacophores, some inhibitors were designed and synthesized to optimize the valuable interactions that could perfectly fit the enzymatic active pockets. Luttens et al.^145^ carried out several screening cycles and combinations of promising scaffolds for SARS‐CoV‐2 3CL^pro^ inhibitors. Compound **19b** was optimized as a noncovalent broad‐spectrum 3CL^pro^ inhibitor and showed promising antiviral activity, as well as good metabolic stability and plasma protein binding in humans.

In addition, some synthetic compounds bear at least reactive groups (such as Michael receptors and α,β‐unsaturated carbonyl) that can covalently bind to crucial residuals (e.g., Cys145) of SARS‐CoV‐2 3CL^pro^, giving rise to irreversible inactivation of the target enzyme.^146^ Such inhibitors generated long‐lasting and efficient inhibitory effects against SARS‐CoV‐2 3CL^pro^, implying a promising strategy for the development of anti‐COVID‐19 agents.^16^, ^147^, ^148^ Jin et al.^16^ screened six inhibitors against SARS‐CoV‐2 3CL^pro^ from a library of 3CL^pro^ containing over 10,000 compounds, and ebselen and PX‐12 could covalently bind to Cys145 of 3CL^pro^, and ebselen might also noncovalently bind to 3CL^pro^ simultaneously. Beyond that, ebselen could react with cysteine residues of several viral proteases, such as SARS‐CoV‐2 PL^pro^ and 3C^pro^ of enterovirus A71 and enterovirus D68, which was suggested as a multi‐target antiviral agent.^149^ Some ebselen and ebsulfur derivatives were synthesized as SARS‐CoV‐2 3CL^pro^ inhibitors, and **1i** and **2k** were proved as potent and covalent inhibitors, with *K_i_* values of 0.031 and 0.078 μM, respectively.^150^, ^151^

Mitsuya and coworkers have been devoted to developing effective 3CL^pro^ inhibitors for fighting SARS‐CoV and SARS‐CoV‐2. In particular, they suggested that carbonyl indole could function as a warhead to modify Cys145 of 3CL^pro^, and several carbonyl‐indole‐containing compounds were identified as covalent inhibitors, such as GRL‐0920.^147^, ^148^, ^152^, ^153^ Another compound, GRL‐1720, an inhibitor of SARS‐CoV 3CL^pro^ (IC_50_ = 30 nM), could also irreversibly inhibit SARS‐CoV‐2 3CL^pro^ (*K*_inact_ = 2.53 min^−1^, *K_i_* = 2.15 μM). Meanwhile, this compound could also block the infectivity of SARS‐CoV‐2^WK‐521^ (SARS‐CoV‐2 JPN/TY/WK‐521 strain) in Vero E6 cells, with an EC_50_ value of 15 μM and an apparent half‐maximal cytotoxicity concentration (CC_50_) value more than 100 μM.^117^, ^148^ Recently, a group of 5‐chloropyridinyl indole carboxylate derivatives was designed for inhibiting SARS‐CoV‐2 3CL^pro^. Among all tested compounds, **7d** was a potent SARS‐CoV‐2 3CL^pro^ inhibitor (IC_50_ = 73 nM) that blocked viral infection in vitro (EC_50_ = 15 μM). The detailed structure–activity relationship (SAR) analysis revealed that the 5‐chloropyridinyl ester was crucial for inhibitory activity. On the indole ring, the N‐allyl substituent could significantly improve the activity, while the incorporation of the methyl group at position‐5 and fluorine at position‐6 generated a declining potency. The X‐ray crystal structure of **7b** and SARS‐CoV‐2 3CL^pro^ presented a covalent binding mechanism.^152^ Subsequently, compound **9a** was identified as a SARS‐CoV‐2 3CL^pro^ inhibitor from another series of new 5‐chloropyridinyl ester analogs, with a calculated IC_50_ value of 160 nM.^153^

Recently, the SAR study of a group of benzoisothiazolone‐containing SARS‐CoV‐2 3CL^pro^ demonstrated that the phenyl group was optimized as the best group at the tail benzene ring, and the acetamide group in the linker was essential to the inhibitory activity. In particular, the crucial benzoisothiazolone that could function as a warhead for covalently binding to Cys145 of 3CL^pro^ should avoid adverse steric hindrance. As shown in Table S2, **16b‐3** was a promising lead compound for novel anti‐COVID‐19 agents.^154^ Beyond that, the thiazolidinone derivatives (k3), QUB‐00006‐Int‐07, VS10, and VS12 were promising inhibitors for SARS‐CoV‐2 3CL^pro^.^155^ ^—^ ^157^ Additionally, it has been reported that metal‐based (such as Au, Pt, and Re) coordination compounds could form metal complexes with cysteine proteases via coordinate covalent bonds.^158^ ^—^ ^160^ Karges et al.^91^ preliminarily suggested that the [Re(2,2′‐bipyridine) (CO)_3_]^+^ fragment could bind to Cys145 of SARS‐CoV‐2 3CL^pro^ with the lowest energy. Then, a series of Re(I) tricarbonyl complexes were synthesized for SARS‐CoV‐2 3CL^pro^ inhibitor evaluation. Both compounds **34** and **22** formed a metal—Cys145 covalent bond with SARS‐CoV‐2 3CL^pro^ in the axial position. Preliminary studies indicate that this compound was a selective inhibitor of 3CL^pro^, which exhibited poor inhibitory effects against several human proteases at 50 μM, including human serine protease dipeptidyl peptidase‐4, aspartate protease β‐secretase 1, and cysteine protease cathepsin B.

## 4 NATURALLY DERIVED SARS‐COV‐2 3CL^pro^ INHIBITORS

It is well known that natural compounds are still the major sources for the identification of drug lead compounds.^161^, ^162^, ^163^, ^164^, ^165^ Over the past few years, a number of structurally diverse natural products and their derivatives (such as flavonoids, phenolic acids, tannins, and quinones) have been found to have anti‐SARS‐CoV‐2 3CL^pro^ effects, and some of them have been identified as covalent 3CL^pro^ inhibitors.^96^, ^166^ In this review, the reported naturally derived SARS‐CoV‐2 3CL^pro^ inhibitors, accompanied by their inhibitory effects and inhibitory mechanisms, were well summarized, which well explained the anti‐COVID‐19 effects of some herbal medicines and provided new inspiration to medicinal chemists for designing and developing novel anti‐COVID‐19 agents by targeting 3CL^pro^.

### 4.1 Flavonoids and their derivatives

**FIGURE 5:**
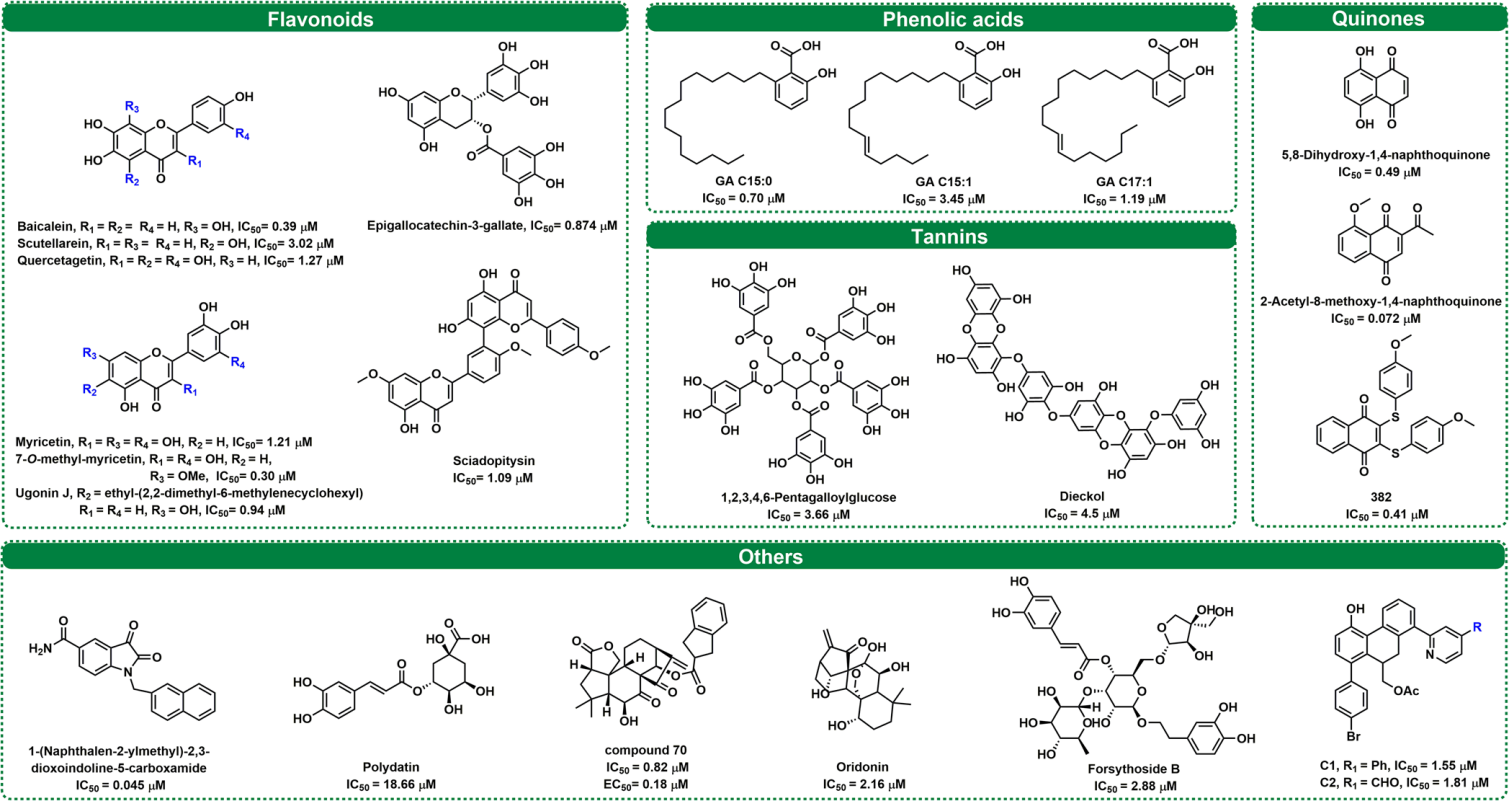
The structures and half‐maximal inhibitory concentration (IC_50_) values for representative naturally derived SARS‐CoV‐2 3CL^pro^ inhibitors

Flavonoids are secondary metabolites that widely exist in edible and medicinal plants and usually comprise several subclasses, such as flavanones, flavones, flavonols, and biflavones.^167^, ^168^ This class of polyphenol compounds is known for good safety profiles and multiple health benefits, including antioxidative, anti‐inflammatory, anticancer, antiviral, and immunomodulatory effects.^169^, ^170^, ^171^, ^172^, ^173^, ^174^ Recently, many flavonoids have shown strong inhibitory effects against SARS‐CoV‐2 3CL^pro^, and their inhibitory effects are listed in Figure 5 and Table S3.

*Scutellaria baicalensis* is a traditional Chinese medicine used for upper respiratory tract infections and possesses wide antiviral activity, including against SARS‐CoV‐2 (EC_50_ = 0.74 μg/ml).^175^, ^176^ Recently, some flavonoids in *S. baicalensis* were reported to be SARS‐CoV‐2 3CL^pro^ inhibitors, such as baicalein and scutellarein.^177^ It is well known that phenolic groups can transform into orthoquinone under oxidizing conditions, which can easily be attacked by nucleophiles (such as thiol).^178^ According to a new study, six scutellarein‐methylated derivatives were synthesized as novel 3CL^pro^ inhibitors. 4′‐*O*‐methylscutellarein was characterized as a potent noncovalent 3CL^pro^ inhibitor (IC_50_ = 0.40 μM). Further SAR study demonstrated that the replacement of hydroxyl groups at the A‐ring was indispensable, and hydrophobicity of the B‐ring might be beneficial to inhibitory activity.^179^ Xiong et al.^96^ identified myricetin, dihydromyricetin, and iso‐dihydromyricetin as covalent inhibitors for SARS‐CoV‐2 3CL^pro^, whose orthoquinone form could modify the key cysteines near the catalytic site (Cys145) or dimeric interface (Cys300) of the target enzyme. Meanwhile, Su et al.^180^ also demonstrated that myricetin could covalently bind to Cys145 of SARS‐CoV‐2 3CL^pro^ by using crystal structure analysis of the complex. To gain more ideal SARS‐CoV‐2 3CL^pro^ inhibitors, several analogs were designed based on myricetin, among which 7‐*O*‐methyl‐dihydropopulins showed the highest inhibitory effect with an IC_50_ value of 0.26 μM. Moreover, this study revealed that the pyrogallol group could be used as an alternative electrophile warhead to develop covalent inhibitors for 3CL^pro^. In contrast, another study revealed that baicalein was a noncovalent inhibitor that could act as a “shield” to prevent the substrate from entering the catalytic pocket.^181^ Even though these compounds bear a pyrogallol group, the different conformations of myricetin and baicalein in the 3CL^pro^ catalytic site generate different action modes.

Ugonin J is a flavonoid isolated from the Rhizome of *Helminthostachys zeylanica*, which has an ethyl‐(2,2‐dimethyl‐6‐methylenecyclohexyl) moiety at the C‐6 position and possessed a potent inhibitory effect on SARS‐CoV‐2 3CL^pro^ (IC_50_ = 0.94 μM). Furthermore, the anti‐SARS‐CoV‐2 activity and anti‐inflammatory activity of this inhibitor have been proven in vitro, suggesting that ugonin J could be used as a leading compound to fight COVID‐19.^182^ Quercetin has a broad spectrum of antiviral activities (including poliovirus type 1, herpes simplex virus type 1 [HSV‐1], HSV‐2, respiratory syncytial virus, and influenza A subtypes).^39^ Regarding anti‐3CL^pro^ activity, quercetin exhibited a better inhibitory effect against SARS‐CoV‐2 3CL^pro^ than SARS‐CoV 3CL^pro^.^166^, ^183^, ^184^, ^185^, ^186^ Recently, Mangiavacchi et al.^187^ synthesized and evaluated a series of compounds based on the skeletons of quercetin and chrysin. The SAR analysis suggested that the phenylselenyl moiety at the C‐8 position was highly effective, while the double substitution resulted in a drop in the activity. 2‐(3,4‐Dihydroxyphenyl)‐3,5,7‐trihydroxy‐8‐(p‐tolylselanyl)‐4H‐chromen‐4‐one was a strong (IC_50_ = 11 μM) and reversible (*K_i_ *= 3.8 μM) 3CL^pro^ inhibitor, presenting a safe and effective inhibition activity (IC_50_ = 8 μM) against the replication of SARS‐CoV‐2 in a Vero cell model. The major tea catechins, including epigallocatechin‐3‐gallate, (‐)‐epicatechin 3‐*O*‐caffeoate, as well as etc‐pyrrolidinone C and D, possessed strong inhibitory effects toward 3CL^pro^.^188^, ^189^, ^190^, ^191^, ^192^ In addition, some flavonoids, such as kaempferol, luteolin, genkwanin, and isorhamnetin, displayed various degrees of inhibitory activities toward 3CL^pro^.^192^, ^193^

However, the bioavailability of naturally occurring flavonoids is generally poor, which are easily glycosylated by UDP‐glucuronosyltransferases in vivo.^194^ The metabolites (glycosyl flavonoids) have better solubility, stability, and bioavailability properties than their aglycones.^193^, ^195^, ^196^ Some molecular docking and simulation studies found that glycosyl flavonoids calculated a high‐affinity score for binding to 3CL^pro^.^197^, ^198^, ^199^, ^200^ By using experimental (spectroscopy and calorimetry) and simulation techniques (docking and molecular dynamics simulations), Rizzuti et al.^202^ revealed that rutin was a promising inhibitor of SARS‐CoV‐2 3CL^pro^.^201^, ^202^ However, most studies found that the introduction of glycosides on flavonoids would weaken the inhibitory effects on 3CL^pro^. For example, baicalin, narcissoside, and kaempferol‐3‐*O*‐gentiobioside presented relatively poorer inhibitory effects than their aglycones.^181^, ^203^ Further SAR studies demonstrated that glycosylation on the 7‐hydroxy of quercetin could be allowed, but the acetoxylation of the glycosyl was adverse.^204^

Biflavones are a class of compounds with a dimer of flavonoid structure, some of which have been validated as 3CL^pro^ inhibitors.^205^ Xiong et al.^166^ found that *Ginkgo biloba* leaves showed strong inhibitory activity against SARS‐CoV‐2 3CL^pro^ via a scale screening of herbal extracts, while 20 major constituents (including five biflavones) isolated from this herb were collected for SARS‐CoV‐2 3CL^pro^ inhibition assays. Further kinetic analyses and molecular docking suggested that sciadopitysin could strongly inhibit SARS‐CoV‐2 3CL^pro^ in a mixed manner, with a *K_i_* value of 2.96 μM. Other biflavones, including ginkgetin, isoginkgetin, amentoflavone, and bilobetin, could also dose dependently inhibit SARS‐CoV‐2 3CL^pro^, with IC_50_ values ranging from 2.33 to 11.19 μM.

### 4.2 Phenolic acids

Phenolic acids are a group of secondary metabolites and bioactive compounds produced by plants.^206^, ^207^ Xiong et al.^166^ pointed out that four ginkgolic acids (GAs) from *Folium ginkgo* showed relatively potent SARS‐CoV‐2 3CL^pro^ inhibitory activity (IC_50_ < 5 μM). Further inhibition kinetic studies and docking simulations clearly showed that GAs **C15:0** and **C17:1** strongly inhibited SARS‐CoV‐2 3CL^pro^ in a mixed manner (Figure 5 and Table S4). Moreover, GAs (**C15:0** and **C15:1**) were identified as dual inhibitors targeting both 3CL^pro^ and PL^pro^ of SARS‐CoV‐2 at nontoxic concentrations by Chen et al.^208^ However, allergenic GAs are severely restricted in commercially available *G. biloba* products.^209^ There is growing evidence that GA has broad antiviral effects by interfering with viral replication.^210^, ^211^ Additionally, Nguyen et al.^212^ reported the inhibitory activity of different phenolic acids from black garlic on SARS‐CoV‐2 3CL^pro^, including gallic acid, caffeic acid, vanillic acid, ferulic acid, and chlorogenic acid. The above results suggest that these phenolic acids are worth exploring as potential new therapeutics for COVID‐19.

### 4.3 Tannins

Tannins displayed potent inhibitory effects against SARS‐CoV‐2 3CL^pro^ and SARS‐CoV 3CL^pro^ with IC_50_ values at the micromolar level. Wang et al.^213^ found tannic acid to be a potent inhibitor of SARS‐CoV‐2 3CL^pro^ and TMPRSS2, with an IC_50_ of 13.4 μM for SARS‐CoV‐2 3CL^pro^. Consistently, tannic acid could also target the mechanisms governing virus entry.^213^ As early as 2005, Chen et al.^215^ demonstrated a significant inhibitory effect of tannic acid on SARS‐CoV 3CL^pro^ (IC_50_ = 3 μM).^190^, ^214^, ^215^ Therefore, the above results suggest that tannic acid has a high potential for the development of anti‐coronavirus therapeutics as a broad‐spectrum inhibitor. As shown in Table S5, 1,2,3,4,6‐pentagalloylglucose is a hydrolysable tannin that has been reported to inhibit a variety of viruses.^216^ In terms of anti‐3CL^pro^ activity, Chiou et al.^188^ found that 1,2,3,4,6‐pentagalloylglucose inhibited 50% of SARS‐CoV‐2 3CL^pro^ and SARS‐CoV 3CL^pro^ at 3.66 and 6.89 μM, respectively.

Additionally, Park et al.^217^ evaluated the biological activity of nine phlorotannins, dieckol (IC_50_ = 2.7 μM), which possesses two eckol groups linked through a diphenyl ether and showed the most potent SARS‐CoV 3CL^pro^ inhibitory activity. Up to now, Yan et al.^218^ developed a novel screening method combining fluorescence polarization technology with a biotin–avidin system and identified dieckol as a new competitive inhibitor against SARS‐CoV‐2 3CL^pro^ with an IC_50_ value of 4.5 μM. Recently, Du et al.^219^ demonstrated that chebulagic acid and punicalagin, which have been recognized as broad‐spectrum antiviral agents, inhibited SARS‐CoV‐2 plaque formation in a dose‐dependent manner, indicating that they exhibit antiviral activity in vitro. Furthermore, chebulagic acid and punicalagin exhibited reversible inhibitory effects against SARS‐CoV‐2 3CL^pro^ via noncompetitive modes.

### 4.4 Quinones and their derivatives

Quinones are a class of cyclohexadienedione‐containing or cyclohexadiene dimethylene‐containing organic compounds that are usually divided into benzoquinones, naphthoquinones, phenanthraquinones, and anthraquinones.^220^, ^221^ As shown in Figure 5 and Table S6, quinones and their derivatives provide several promising leading compounds for the development of anti‐COVID‐19 agents by targeting SARS‐CoV‐2 3CL^pro^. It has been reported that tanshinones isolated from *Salvia miltiorrhiza* are inhibitors of SARS‐CoV cysteine proteases (including 3CL^pro^ and PL^pro^).^222^ Recently, tanshinone I was shown to inhibit SARS‐CoV‐2 at the cellular level with an EC_50_ value of 2.26 μM. These results substantiate the use of tanshinone derivatives as antiviral agents. In the meantime, tanshinone I and tanshinone IIA were identified as SARS‐CoV‐2 PL^pro^ inhibitors.^223^, ^224^, ^225^ Jin et al.^16^ found that shikonin exhibits potent inhibition of SARS‐CoV‐2 3CL^pro^ activity with an IC_50_ of 15.75 μM. Moreover, shikonin presented a noncovalent binding configuration with multiple interactions at the S1–S4 subsites of the binding pocket and occupied the space of one water molecule of 3CL^pro^.^56^

It is well known that the specific chemical structure of quinone confers oxidative and electrophilic properties.^220^, ^226^, ^227^, ^228^ Wang et al.^229^ screened vitamin K3 as a time‐dependent SARS‐CoV‐2 3CL^pro^ inhibitor (IC_50_ = 4.78 μM at 60 min preincubation) from Food and Drug Administration (FDA)‐approved drug library. Based on this finding, a set of vitamin K3 analogs was collected for SAR analysis. The results showed that 5,8‐dihydroxy‐1,4‐naphthoquinone could strongly and time dependently inhibit SARS‐CoV‐2 3CL^pro^ and covalently bind to the target enzyme. However, the high electrophilicity of quinones may lead to cytotoxicity.^230^ To discover safe and effective quinone‐derived inhibitors against SARS‐CoV‐2 3CL^pro^, Cui and Jia^231^ designed a set of juglone‐like compounds by using a simple skeleton. The results suggested that the interaction between the acetyl substituent on the quinone ring and the methyl group attached to the phenolic hydroxyl group of juglone was crucial for the inhibitory effects. Further cytotoxicity and antiviral assays demonstrated that 2‐acetyl‐8‐methoxy‐1,4‐naphthoquinone exhibited a low cytotoxic profile and good anti‐SARS‐CoV‐2 in Vero E6 cells (EC_50_ = 4.55 μM). This study demonstrated the possibility of quinone being developed as a safe antiviral agent. Recently, four compounds were screened to inhibit SARS‐CoV‐2 3CL^pro^ from a compound library, with IC_50_ values ranging from 0.41 to 66 μM. Further studies suggested that compound **382** was a reversible SARS‐CoV‐2 3CL^pro^ inhibitor, while compound **415** might form a covalent bond with Cys145 of 3CL^pro^.^232^ Similarly, aloesin was identified as a SARS‐CoV‐2 3CL^pro^ inhibitor from a fluorescence resonance energy transfer (FRET)‐based THS assessment.^233^

### 4.5 Others

Beyond the abovementioned classes of natural compounds, other compounds derived from natural compounds were also found to have SARS‐CoV‐2 3CL^pro^ inhibition activity, such as alkaloids, terpenoids, and theaflavins, as well as phenylethanol glycosides.^181^, ^214^, ^234^, ^235^ The compound information alongside their SARS‐CoV‐2 3CL^pro^ inhibitory effects are shown in Figure 5 and Table S7. Some isatin derivatives exhibited strong inhibition effects against 3CL^pro^, such as 1‐(naphthalen‐2‐ylmethyl)‐2,3‐dioxoindoline‐5‐carboxamide, which was a promising compound for developing broad‐spectrum anti‐coronavirus agents.^236^, ^237^ Zhong et al.^238^ identified that oridonin displayed effective inhibition of SARS‐CoV‐2 3CL^pro^ activity and bound to 3CL^pro^ via covalent bonding, while inhibiting SARS‐CoV‐2 in Vero E6 cells with an IC_50_ of 4.95 μM, above indicating that oridonin prevented SARS‐CoV‐2 replication by inhibiting 3CL^pro^.^239^ Most recently, by pharmacophore‐oriented semisynthesis combining the pharmacophore of oridonin and a novel scaffold (maoelactone A), Zhou et al.^240^ created a series of compounds with anti‐SARS‐CoV‐2 activity, where compound **70** inhibited the replication of SARS‐CoV‐2‐affected Vero E6 cells with low EC_50_ values.

One newly reported study indicated that six phenylethanol glycosides (including forsythoside A, B, E, H, I, and isoforsythiaside) isolated from *Forsythia suspensa* were strong inhibitors of SARS‐CoV‐2 3CL^pro^ (IC_50_ values range from 2.88 to 10.17 μM), which contributed to the excellent anti‐SARS‐CoV‐2 activity of Shuanghuanglian preparation (a traditional proprietary Chinese medicine).^181^ Some other compounds, including polydatin, resveratrol, and all‐*trans* retinoic acid, generated inhibitory effects against SARS‐CoV‐2 3CL^pro^.^241^, ^242^ A series of 9,10‐dihydrophenanthrene derivatives were synthesized to discover strong SARS‐CoV‐2 3CL^pro^ inhibitors.^243^ The preliminary SAR suggested that a suitable bulkier group at the C‐8 position displayed good inhibitory activities. Among all derivatives, compound **C1** could dose dependently inhibit the target enzyme in a mixed manner, with an IC_50_ value of 1.55 μM and a *K_i_* value of 6.09 μM. Further study suggested that this inhibitor had the potential to be a novel orally administered and broad‐spectrum antiviral agent.^243^

## 5 ANTI‐COV CLINICAL CANDIDATES TARGETING SARS‐COV‐2 3CL^pro^

### 5.1 SARS‐CoV‐2 3CL^pro^ inhibitors under clinical trials

**FIGURE 6:**
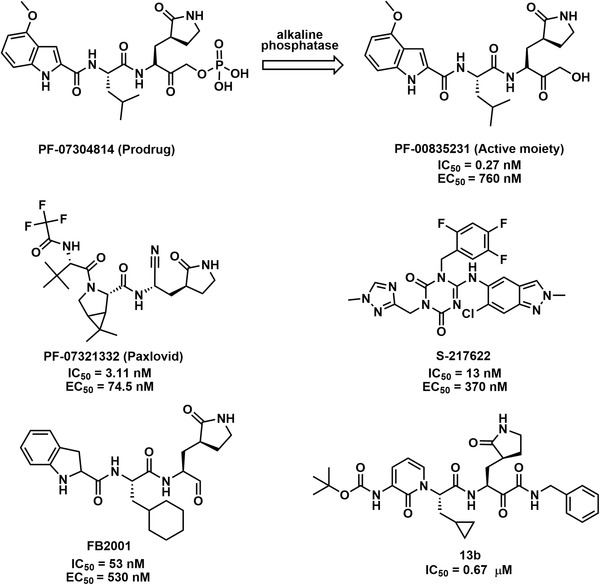
The structures and half‐maximal inhibitory concentration (IC_50_) values for representative clinical candidates SARS‐CoV‐2 3CL^pro^ inhibitors, as well as their half‐maximal effect concentration (EC_50_) values for anti‐SARS‐CoV‐2

In view of the ongoing mutation of SARS‐CoV‐2 (such as Omicron), clinical studies of some antibody drugs have stagnated.^17^, ^244^ Small‐molecule anti‐CoV drugs, however, have great potential to combat new CoV variants, as the convenience and flexibility of oral administration, along with the large production capacity, provide good conditions to achieve a global fight against COVID‐19.^245^ The following are some clinical advances in the development of small‐molecule drugs targeting SARS‐CoV‐2 3CL^pro^ (Figure 6 and Table 2).^25^, ^78^, ^246^, ^247^, ^248^, ^249^, ^250^, ^251^, ^252^, ^253^, ^254^, ^255^

**TABLE 2:** Representative clinical candidates for SARS‐CoV‐2 3CL^pro^ inhibitors

Drug	Company	Delivery	States	IC_50_ (nM)	EC_50_ (nM)	CT.GOV ID/Ref.
PF‐07321332 (Paxlovir)	Pfizer	Oral	Proved	3.11	74.5	NCT04960202^258^
s‐217622	Shionogi	Oral	Phase III	13	370	NCT05305547^260^
PF‐07304814	Pfizer	IV	Phase I	0.27	760	NCT05050682
FB2001/11a (DC402234)	Frontier	IV	Phase I	53	530	NCT05197179
EDP‐235	Enanta	Oral	Phase I	5.8	5.1	NCT05246878
SIM0417 (SSD8432)	Simcere	Oral	Phase II	–	–	NCT05373433
PBI‐0451	Pardes	Oral	Phase I	–	–	NCT05011812
13b	University of Lübeck	Inhaled	Preclinical	670	4–5 μM (Calu‐3 cell)	^25^
ALG‐097111	Aligos	–	Preclinical	7	200 (A549 cell)	^251^
MPI8	Sorrento	–	Preclinical	105	30	^134^
ASC11	Ascletis	Oral	Preclinical	–	–	^263^
EDDC‐2214	Everest	Oral	Preclinical	–	–	^264^
RAY003	Zhongsheng	Oral	Preclinical	–	–	^179^

Abbreviations: EC_50_, half‐maximal effect concentration; IC_50_, half‐maximal inhibitory concentration.

Recently, paxlovid, a novel orally available agent combining a 3CL^pro^ inhibitor (PF‐07321332) with ritonavir, has been approved by the FDA for the treatment of patients with moderate or severe COVID‐19.^247^, ^248^, ^256^, ^257^, ^258^ PF‐07321332 exhibited potent inhibition against 3CL^pro^ from various CoV types known to infect humans, as well as significant SARS‐CoV‐2 antiviral activity in Vero E6 cells (EC_50_ = 74.5 nM). In the meantime, PF‐07321332 (100 μM) showed no inhibitory activity against caspase‐2, cathepsin B/D/L, chymotrypsin, elastase, thrombin, and HIV‐1 protease, indicating a high selectivity for CoV proteases. A phase II/III clinical trial of PF‐07321332/ritonavir (ID: NCT04960202) assessed its safety and effectiveness for treating COVID‐19 in adults who did not require hospitalization. The data showed that the patients treated with this drug experienced an 89% reduction in the risk of hospitalization or death, which was highly effective. More recently, the clinical trial results from the treatment of hospitalized patients with paxlovid oral agents during the Omicron BA.2 outbreak in Hong Kong showed significantly lower disease progression composite outcomes (hazard ratio [HR] = 0.33, *p* < 0.001), significantly lower all‐cause mortality (HR = 0.32, *p* < 0.001), and faster reduction in viral load (HR = 1.25, *p* = 0.015).^259^ Additionally, the novel phosphate prodrug PF‐07304814, which can be rapidly converted in vivo to the active moiety of PF‐00835231, has broad‐spectrum inhibitory activity against a panel of 3CL^pro^ and potent antiviral activity in vivo.^253^, ^254^ Furthermore, clinical trials in phase Ib of PF‐07304814 (ID: NCT05050682) evaluated its safety, metabolism, and pharmacokinetics in patients with SARS‐CoV‐2 infection.

After virtual screening and SAR analysis of the hit compounds, S‐217622 displayed potent inhibition activity against SARS‐CoV‐2 3CL^pro^ and exhibited in vitro antiviral activity against a range of CoVs, including more aggressive SARS‐CoV‐2 variants.^249^, ^250^, ^252^ The clinical trial of S‐217622 (ID: NCT05305547) was a randomized, placebo‐controlled, double‐blind study with Japanese adults, which evaluated the antiviral effects and safety of this drug once daily for 5 days. New data indicated that the proportion of patients with positive viral titers was decreased by approximately 90% versus placebo on the fourth day of treatment.^260^ Dai et al.^78^ based on the crystal structure of 3CL^pro^ designed and synthesized peptidomimetic compounds **11a** and **11b**, which possess potent antiviral activity with EC_50_ values of 530 and 720 nM against SARS‐CoV‐2, respectively. Of these, FB2001 (**11a**) was an anti‐CoV candidate for reaching clinical trials. In addition, FB2001 (ID: NCT05197179) demonstrated excellent safety and tolerability in the first human clinical trial conducted in the United States, for which its pharmacokinetics and safety will subsequently be evaluated in healthy Chinese populations.

At the 2022 Annual Meeting of American Society for Biochemistry and Molecular Biology, Enanta noted that EDP‐235 potently inhibited the SARS‐CoV‐2 3CL^pro^ protease and effectively blocked the replication of SARS‐CoV‐2 in multiple cellular models. In addition, EDP‐235 was shown to have good in vivo penetration into a variety of target tissues.^246^ Furthermore, EDP‐235 (ID: NCT05246878) was evaluated in the first in‐human phase I study in healthy volunteers for safety, tolerability, and pharmacokinetics. PBI‐0451 (ID: NCT05011812), administered twice daily as a stand‐alone agent, has shown good tolerability in the ongoing phase I clinical trial, at >20‐fold single‐ and >14‐fold multiple‐total daily dosage.^261^ SSD8432 (ID: NCT05373433) was the first oral SARS‐CoV‐2 3CL^pro^ drug approved for clinical trials in China, and its phase II clinical trial evaluated its efficacy and safety in combination with ritonavir in asymptomatic infections or mild/general safety studies in adult subjects with COVID‐19. The role of an α‐ketoamide inhibitor was explored, and it was found that compound **13b** could inhibit 3CL^pro^ from SARS‐CoV‐2, SARS‐CoV, and MERS‐CoV, with IC_50_ values of 0.67, 0.90, and 0.58 μM, respectively.^25^ The inhibitory effect of compound **13b** on human Calu‐3 cells infected with SARS‐CoV‐2 (EC_50_ = 4–5 μM). Furthermore, the pharmacokinetic profile of the optimized inhibitor revealed a clear pulmonary propensity and was suitable for administration via the inhalation route.

In brief, 3CL^pro^ inhibitor therapy is an attractive and effective pharmacotherapy for treating CoV‐associated infectious diseases, owing to its broad spectrum of antiviral activities and ability to prevent the posttranslational processing of SARS‐CoV‐2 polypeptides as well as reduce the risk of mutation‐mediated resistance to drug therapy.^262^

### 5.2 Old drugs as SARS‐CoV‐2 3CL^pro^ inhibitors

It is well known that the development of a novel drug generally takes a long time. Comprehensive clinical studies of approved drugs promote drug repurposing a shortcut for the discovery of safe and effective anti‐COVID‐19 agents, which can bypass animal safety studies and directly enter clinical phase II or III to ensure supply. Many approved drugs have been identified as SARS‐CoV‐2 3CL^pro^ inhibitors by using computational and experimental studies, such as teicoplanin, dipyridamole, hydroxychloroquine, and chloroquine,^265^, ^266^ and their inhibitory effects are listed in Table 3.

**TABLE 3:** The current indications of clinical drugs and their inhibitory activities against SARS‐CoV‐2 3CL^pro^

Compound	Pharmacological activities	IC_50_/*K_i_* (μM)	Ref.
Teicoplanin	Antibacteria	1.61	^265^, ^273^
Dipyridamole	Antiplatelet	0.04	^265^
Hydroxychloroquine	Antimalarial and anti‐inflammatory	0.36	
Chloroquine	Antimalarial and anti‐inflammatory	0.56	
Manidipine	Anti‐hypertension	4.81	^141^
Lercanidipine	Anti‐hypertension	16.2	
Efonidipine	Anti‐hypertension	38.5	
Bedaquiline	Antituberculosis	18.7	
Ethacrynic acid	Hydragogue for treating chronic heart failure	1.11	^267^, ^274^
Naproxen	Nonsteroidal anti‐inflammatory drug for treating mild‐to‐moderate pain and arthritis	3.45	^267^, ^275^
Allopurino	Treat gout, hyperuricemia, and kidney stones	3.77	^267^, ^276^
Butenafine hydrochloride	Antifungal	5.40	^267^, ^277^
Raloxifene hydrochloride	Prevent osteoporosis	5.61	^267^, ^278^
Tranylcypromine hydrochloride	Antidepressant and antianxiety	8.64	^267^, ^279^
Saquinavir mesylate	Anti‐HIV	9.92	^267^, ^280^
Triptorelin acetate	Anti‐prostate cancer	10.12	^267^, ^281^
Goserelin acetate	Anti‐prostate and breast cancer	12.02	^267^, ^282^
Rocuronium bromide	Muscle relaxant	17.47	^267^, ^283^
Bisacodyl	Treat constipation	17.51	^267^, ^284^
Armodafini	Promotes wakefulness	17.87	^267^, ^285^
Clobetasol propionate	Treat skin conditions	18.09	^267^, ^286^
Sirolimus (Rapamycin)	An immunosuppressant drug for allografting rejection therapy	22.30	^267^, ^287^
Colistin sulfate	Antibacteria	23.20	^267^, ^273^
Cetirizine	Relieve allergy	25.58	^267^, ^288^
Bexarotene	Treat cutaneous T‐cell lymphoma	26.49	^267^, ^289^
Cefpodoxime proxetil	Antibacteria	32.43	^267^, ^290^
Clindamycin palmitate hydrochloride	Antibacteria	33.21	^267^, ^291^
Oxaliplatin	Anti‐colorectal cancer	47.31	^267^, ^292^
Masitinib	Inhibit tyrosine kinase	2.5/2.6	^268^, ^293^
Colloidal bismuth subcitrate	Anti‐*Helicobacter pylori*, anti‐duodenal ulcer	0.93	^94^, ^97^
Merbromin	Antibacteria	2.7	^270^, ^295^
Tolcapone	Treat Parkinson's disease	7.9	^271^
Levothyroxine	Thyroid hormone	19.2	
Manidipine‐2HCl	Anti‐hypertension	10.4	
Disulfiram	Alcohol aversion	9.35	^16^, ^296^
Carmofur	Antitumors	1.82	^16^, ^297^
Tideglusib	Anti‐Alzheimer disease	1.55	^16^, ^298^
Z‐FA‐FMK	Inhibit cysteine proteases irreversibly	26.3	^141^, ^272^
Boceprevir	Anti‐HCV protease	5.40	^272^, ^299^

Abbreviations: HCV, hepatitis C virus; HIV, human immunodeficiency virus; IC_50_, half‐maximal inhibitory concentration; *K_i_*, inhibition constant.

According to the predicted poses and docking scores of complexes, 17 agents were predicted as potential inhibitors for SARS‐CoV‐2 3CL^pro^, five of which could inhibit the hydrolysis of SARS‐CoV‐2 3CL^pro^‐catalyzed fluorescent peptide substrate.^141^ Chiou et al.^267^ identified 20 drugs as SARS‐CoV‐2 3CL^pro^ inhibitors in silico and in vitro. Among them, ethacrynic acid was the strongest inhibitor, with an IC_50_ value of 1.11 μM, while the anti‐inflammatory and immunosuppressive activities of naproxen (IC_50_ = 3.45 μM) might be advantageous in COVID‐19 treatment. A large‐scale screening campaign was conducted for the anti‐OC43 (one β‐CoV) effects in vitro. Twenty drugs were screened out for anti‐SARS‐CoV‐2 infection assays in A549 cells and enzyme measurements in green fluorescent protein (GFP)‐expressing 293T cells. Masitinib competitively inhibited SARS‐CoV‐2 3CL^pro^, both in vitro and in live cells. Moreover, this agent also significantly reduced the SARS‐CoV‐2 viral load in mice and inflammatory cytokines in the lungs. The clinical combination of masitinib and isoquercetin suggested that masitinib was a promising agent for the early treatment of COVID‐19.^268^

CBS is a metallodrug usually used for duodenal ulcer treatment. A newly reported study found that CBS remarkably inhibited SARS‐CoV‐2 3CL^pro^ activity in vitro and in cellulo. Rather than the active residual (Cys145), CBS bound to the allosteric site (Cys300) and caused dimeric enzyme to dissociate into monomers.^95^ Additionally, CBS exhibited potent anti‐SARS‐CoV‐2 activity both in the cells and golden Syrian hamster model, which also inactivated SARS‐CoV‐2 helicase by displacing the zinc(II) ions in helicase by bismuth(III) ions. All these findings suggested that CBS was a promising agent for anti‐COVID‐19.^269^ Recently, merbromin was identified as a mixed inhibitor against SARS‐CoV‐2 3CL^pro^.^270^ Manidipine‐2HCl served as a dual inhibitor for SARS‐CoV‐2 3CL^pro^ (IC_50_ = 7.90 μM) and PL^pro^ (IC_50_ = 14.20 μM), showing effective anti‐SARS‐CoV‐2 activity with an EC_50_ value of 14.5 μM.^271^ However, due to the rigorous experimental conditions of cell‐based assays, the majority of reported SARS‐CoV‐2 3CL^pro^ inhibitors were restricted to in vitro effects. In a new study, a novel cell‐based luciferase complementation reporter assay was reported for the discovery of SARS‐CoV‐2 3CL^pro^ inhibitors, which could readily differentiate false positives caused by cytotoxicity. The method was further applied to the validation of cell‐based inhibitory effects and cytotoxicity for 22 reported SARS‐CoV‐2 3CL^pro^ inhibitors.^272^

## 6 CONCLUSIONS AND PERSPECTIVES

The ongoing COVID‐19 pandemic has created a serious threat to human health and life safety worldwide, thus, there is an urgent medical need to find more effective therapeutic strategies for combating COVID‐19.^300^, ^301^, ^302^, ^303^ Among all validated targets for fighting CoVs, including SARS‐CoV‐2, the highly conserved 3D structure of 3CL^pro^ plays an essential role in CoV replication, and no known human protease possesses a similar cleavage specificity, making 3CL^pro^ an ideal target for developing clinically effective anti‐SARS‐CoV‐2 agents.^21^, ^60^, ^109^, ^304^, ^305^ It is worth noting that a wide range of compounds have been found to have strong to moderate SARS‐CoV‐2 3CL^pro^ inhibitory effects in the past few years. To better understand the structural features of SARS‐CoV‐2 3CL^pro^ inhibitors and their inhibitory mechanisms, this study systematically summarized the reported structurally diverse SARS‐CoV‐2 3CL^pro^ inhibitors (including marketed drugs and other synthetic compounds, herbal constituents, and their derivatives), as well as their inhibition potentials and mechanisms of action. The information and knowledge presented here offer a basic reference for medicinal chemists to design and develop more effective 3CL^pro^ inhibitors as novel anti‐SARS‐CoV‐2 agents.

Targeting the key amino acids surrounding the catalytic site to block the hydrolytic process of 3CL^pro^ is one of the practical strategies for developing efficacious 3CL^pro^ inhibitors.^30^, ^31^, ^34^, ^36^, ^37^, ^133^ According to the different inhibitory mechanisms, all reported SARS‐CoV‐2 3CL^pro^ inhibitors can be divided into reversible inhibitors and covalent inhibitors. Most reversible inhibitors of SARS‐CoV‐2 3CL^pro^ exhibit micromolar activity, and these agents have difficulty blocking the hydrolytic activity of 3CL^pro^ in vivo. In contrast, covalent SARS‐CoV‐2 3CL^pro^ inhibitors bear at least one of the warheads (such as pyrogallol groups, quinones, or Michael receptors), which are capable of inhibiting viral replication by forming a covalent bond with the thiol of key cysteines on the catalytic site and dimeric interface of 3CL^pro^ (such as Cys145, Cys300, and Cys44). Compared to reversible inhibitors, these covalent inhibitors tend to show stronger and prolonged activities, which motivates medicinal chemists to develop more potent covalent inhibitors against 3CL^pro^. Although a number of synthetic compounds and natural compounds have been identified as SARS‐CoV‐2 3CL^pro^ covalent inhibitors, most of them show poor bioavailability, poor metabolic stability, and poor aqueous solubility. In the future, the anti‐SARS‐CoV‐2 3CL^pro^ potency and drug‐likeness properties should be improved simultaneously to overcome these limitations.^306^

Another alternative potential strategy for designing SARS‐CoV‐2 3CL^pro^ inhibitors is to block the formation of 3CL^pro^ dimers, the active form of this key enzyme.^307^, ^308^ Considering that the hydrolytic activity of 3CL^pro^ relies on its dimeric form, inhibitors targeting protein self‐association that disturb dimerization formation and stabilization by destroying the key interactions essential for 3CL^pro^ are also highly desirable. Such agents can prevent virus replication and proliferation in the invisible battlefield against this enigmatic and rapidly evolving virus. It has been reported that some known SARS‐CoV‐2 3CL^pro^ inhibitors can bind to either the catalytic site or the allosteric sites (especially the dimer interface) via different binding modes, including competitive, noncompetitive, and mixed manners. Theoretically, it is more likely to block SARS‐CoV‐2 3CL^pro^ by using combinations of various 3CL^pro^ inhibitors that target different ligand‐binding sites, which may display synergistic 3CL^pro^ inhibitory effects via different inhibitory modes (such as occupying the catalytic domain and blocking dimerization formation).

In addition to 3CL^pro^ inhibition activity, many synthetic agents and herbal constituents (including flavonoids, alkaloids, and polyphenols) have been found to have strong inhibitory or modulatory effects on other key targets for treating CoVs (such as PL^pro^, RNA‐dependent RNA polymerase [RdRp], and TMPRSS2).^125^, ^173^, ^309^, ^310^, ^311^, ^312^ It is well known that herbal medicines contain numerous compounds, while various constituents may interact with different anti‐CoV targets or different ligand‐binding sites.^313^, ^314^ In these cases, the synergetic effects of multiple components from herbal medicines should be carefully investigated, which may partially explain the excellent anti‐COVID‐19 activities of some marketed Chinese medicines.^314^ Furthermore, cathepsin L (a lysosomal cysteine protease in the host that cleaves furin‐induced SARS‐CoV‐2 S protein into smaller fragments and activates its membrane fusion) has also been identified as a key target participating in SARS‐CoV‐2 infection.^112^, ^123^, ^315^, ^316^ Thus, it is highly recommended to develop more efficacious dual inhibitors by targeting both viral protease and host cathepsin L to combat COVID‐19.^125^

COVID‐19 is a complex, multi‐organ, and heterogeneous illness, and severe disease cases are frequently accompanied by a hypercoagulable inflammatory state.^317^, ^318^ Thus, an ideal anti‐COVID‐19 medication should have multiple pharmacological activities, such as anti‐inflammatory, anticoagulant, anti‐CoV, and immunomodulatory activities. Numerous studies have confirmed that several marketed Chinese medicines display significant anti‐inflammatory and immunomodulatory effects in vivo, achieving good protective effects on the organs as well as inhibiting viral replication.^319^, ^320^ For example, Qingfei Paidu Decoction, a widely used Chinese medicine prescription for the treatment of COVID‐19 in China, has been found to have multiple pharmacological activities, including anti‐inflammatory, immunomodulatory, and antiviral effects.^321^, ^322^, ^323^, ^324^, ^325^ In the future, to obtain better therapeutic effects, the clinically used Chinese medicine prescriptions for treating COVID‐19 can be used in combination with marketed anti‐CoV agents for clinical observations in a reasonable dose range,^301^ which may be beneficial to COVID‐19 patients with pre‐existing diseases (e.g., cardiovascular disease, diabetes, and pulmonary disease).

## CONFLICTS OF INTEREST

The authors declare they have no conflicts of interest.

## AUTHOR CONTRIBUTIONS

Q.H., Y.X., and GH.Z. drafted this manuscript and prepared the figures. YN.Z. and YW.Z. participated in the collection of the related literature. P.H. and GB.G. supervised the review process. All authors have read and approved the final manuscript.

## ETHICS STATEMENT

No ethical approval is required.

## Supporting information

Supporting information

## Data Availability

All data are freely available from the corresponding author upon request.
