# Supplementary material for: The SARS‐CoV‐2 main protease (Mpro): Structure, function, and emerging therapies for COVID‐19
Source: MedComm (2020). 2022 Jul 14;3(3):e151. doi: 10.1002/mco2.151 (PMC9283855; doi:10.1002/mco2.151)
Supplement: Supplementary file 1 — Supporting information [file MCO2-3-0-s001.docx]

**Supplementary Materials**

*for*

**[The SARS-CoV-2 main protease](https://pubmed.ncbi.nlm.nih.gov/32738988/) (M^pro^): structure, function and emerging therapies for COVID-19**

Qing Hu^a,b,1^, Yuan Xiong^a,1^, Guang-Hao Zhu^a^, Ya-Ni Zhang^a^, Yi-Wen Zhang^b^, Ping Huang^b*^, Guang-Bo Ge^a^^*^

*a. Shanghai Frontiers Science Center of TCM Chemical Biology, Institute of Interdisciplinary Integrative Medicine Research, Shanghai University of Traditional Chinese Medicine, Shanghai, 201203, China*

*b. Clinical Pharmacy Center, Cancer Center, Department of Pharmacy,* *Zhejiang Provincial People's Hospital, Affiliated People's Hospital, Hangzhou Medical College, Hangzhou, Zhejiang, 310014, China*

^∗^ Corresponding authors

E-mail addresses: geguangbo@dicp.ac.cn (G.-B. Ge) & huangping@hmc.edu.cn (P. Huang)

^1^ Contributed equally.

This file contains **seven** supplementary tables.

**Contents**

**Table S1.** The five sub-pockets and key facial residues in the catalytic site of 3CL^pro^.

**Table S2.** The inhibitory effects of peptidomimetic and non-peptidomimetic compounds against SARS-CoV-2 3CL^pro^.

**Table S3.** The inhibitory effects of flavonoids and their derivatives against SARS-CoV-2 3CL^pro^.

**Table S4.** The inhibition potency of phenolic acids against SARS-CoV-2 3CL^pro^.

**Table S5.** The inhibition potency of tannins against SARS-CoV-2 3CL^pro^.

**Table S6.** The inhibition potency of quinones and their derivatives against SARS-CoV-2 3CL^pro^.

**Table S7.** The inhibition potency of other plant-derived compounds against SARS-CoV-2 3CL^pro^.

**Table S1.** The five sub-pockets and key facial residues in the catalytic site of 3CL^pro^.

| **Sub-pocket** | **Position** | **Facial residues** | **Substrate** **specificity** |
| --- | --- | --- | --- |
| S1 | P1 | Phe140, Gly143, Ser144, Cys145, His163, Glu166, His172 | Glutamine, lactam |
| S2 | P2 | Thr25, His41, Cys145 | Leu, Phe, Met, Val |
| S3 | P3 | His41, Met49, Met165 | No specificity |
| S4 | P4 | Met165, Glu166 | Hydrophobic amino acids |
| S5 | P5 | Glu166, Met165, Gln189 | Hydrophobic amino acids |

**Table S2.** The inhibitory effects of peptidomimetic and non-peptidomimetic compounds against SARS-CoV-2 3CL^pro^.

| **Compd.** | **Structure** | **IC_50_ / *K_i_* (μM)** | **Ref.** |
| --- | --- | --- | --- |
| 14a |  | 0.42 | [130] |
| 16a |  | 0.41 |  |
| SDZ224015 | **** | 0.03 | [132] |
| MPI3 |  | 0.0083 | [133] |
| MPI8 |  | 0.105 |  |
| Azanitrile 8 |  | 0.024 | [131] |
| Pyridyl ester 17 |  | 0.01 |  |
| (*R*,*R*)-18 |  | 0.056 | [135] |
| 21a |  | 1.6 | [137] |
| Z236230776 |  | 0.80 | [136] |
| Z1244904919 |  | 0.73 |  |
| Z225729516 | **** | 1.72 |  |
| Z108564100 | **** | 0.76 |  |
| Z106460362 | **** | 2.05 |  |
| 21b | **** | 0.018 | [142] |
| PX-12 |  | 21.39 | [149] |
| 1i | **** | 0.074 | [154] |
| 2k | **** | 0.11 |  |
| 16b-3 |  | 0.116 |  |
| k3 |  | 0.010 | [155] |
| VS10 | **** | 0.20 | [157] |
| VS12 | **** | 1.89 |  |
| 22 |  | 13.8 | [91] |
| 39 |  | 9.6 |  |
| 2j |  | 0.75 | [255] |

**Table** **S3.** The inhibitory effects of flavonoids and their derivatives against SARS-CoV-2 3CL^pro^.

| **Compd.** | **Structure** | **IC_50_ /*K_i_* (μM)** | **Ref.** |
| --- | --- | --- | --- |
| Baicalin |  | 6.41 | [181,203] |
| 4’-*O*-methylscutellarein |  | 0.40 | [179] |
| 6,7-dihydroxy-5-methoxy-2-(4-methoxyphenyl) chromone |  | 16.65 |  |
| Dihydromyricetin |  | 4.91 | [96,180] |
| Iso-dihydromyricetin |  | 3.73 |  |
| Taxifolin |  | 72.72 |  |
| Myricitrin |  | 14.22 |  |
| 7-*O*-methyl-dihydromyricetin |  | 0.26 | [180] |
| Myricetin-7-yl diphenyl phosphate |  | 3.13 |  |
| Dihydromyricetin-7-yl diphenyl phosphate |  | 1.84 |  |
| Kaempferol |  | 21.7 | [166] |
| Isokaempferide |  | 40.23 |  |
| Luteolin |  | 74.86 / 20.48 | [192,193] |
| Quercetin |  | 12.65 / 7.0 | [166,183] |
| Genkwanin |  | 10.62 | [166] |
| Isorhamnetin |  | 31.59 |  |
| Apigenin |  | 153.0 |  |
| 2-(3,4-Dihydroxyphenyl)-3,5,7-trihydroxy-8-(phenylselanyl)-4H-chromen-4-one |  | 5.1 / 1.8 | [179] |
| 2-(3,4-Dihydroxyphenyl)-3,5,7-trihydroxy-8-(butylselenyl)-4H-chromen-4-one |  | 24 / 8.6 |  |
| 2-(3,4-Dihydroxyphenyl)-3,5,7-trihydroxy-8-(p-tolylselanyl)-4H-chromen-4-one |  | 11 / 3.8 |  |
| 2-(3,4-Dihydroxyphenyl)-3,5,7-trihydroxy-8-((4-methoxyphenyl)selanyl)-4*H*-chromen-4-one |  | 3.0 / 1.1 |  |
| 2-(3,4-Dihydroxyphenyl)-3,5,7-trihydroxy-6,8-bis(phenylselanyl)-4H-chromen-4-one |  | 13 / 4.6 |  |
| 5-Hydroxy-2-phenyl-7-[2-(phenyltellanyl)ethoxy]-4H-chromen-4-one |  | 3.3 / 1.1 |  |
| 7-{2-[(4-Chlorophenyl)tellanyl]ethoxy}-5-hydroxy-2-phenyl-4H-chromen-4-one |  | 2.2 / 0.77 |  |
| (-)-Epicatechin 3-*O*-caffeoate |  | 1.58 | [192] |
| Etc-pyrrolidinone C and D |  | 0.90 |  |
| Rutin |  | 31 | [201] |
| Narcissoside |  | 38.142 | [203] |
| Kaempferol-3-*O*-gentiobioside |  | 35.892 |  |
| Vicenin-2 |  | 38.856 |  |
| Isoschafoside |  | 30.220 |  |
| Ginkgetin |  | 2.98 | [166] |
| Isoginkgetin |  | 2.33 |  |
| Amentoflavone |  | 8.65 |  |
| Bilobetin |  | 11.19 |  |

**Table S4.** The inhibition potency of phenolic acids against SARS-CoV-2 3CL^pro^.

| **Compd.** | **Structure** | **IC_50_ (μM)** | **Ref.** |
| --- | --- | --- | --- |
| Ginkgolic acid C13:0 |  | 3.57 | [166] |
| Chlorogenic acid |  | 140 | [212] |
| Caffeic acid |  | 197 |  |

**Table S5.** The inhibition potency of tannins against SARS-CoV-2 3CL^pro^.

| **Compd.** | **Structure** | **IC_50_ (μM)** | **Ref.** |
| --- | --- | --- | --- |
| Tannic acid | 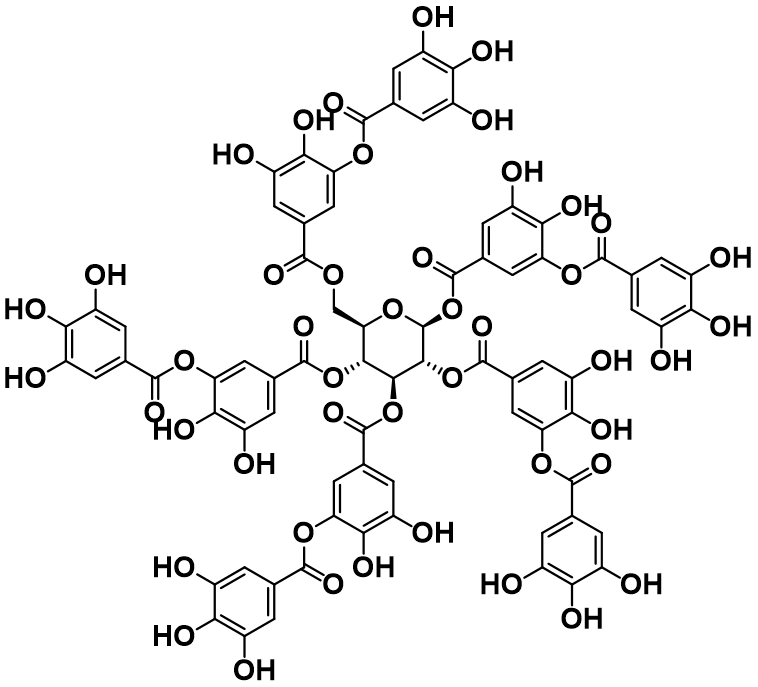 | 13.4 | [213] |
| Chebulagic acid |  | 9.09 | [219] |
| Punicalagin |  | 4.62 |  |

**Table S6.** The inhibition potency of quinones and their derivatives against SARS-CoV-2 3CL^pro^.

| **Compd.** | **Structure** | **IC_50_ (μM)** | **Ref.** |
| --- | --- | --- | --- |
| Shikonin |  | 15.75 | [16] |
| Vitamin K3 |  | 4.78 | [229] |
| 1,4-Naphthoquinone |  | 0.69 / 0.11 |  |
| 2-Chloro-1,4-naphthoquinone |  | 1.92 | [230] |
| 5-Hydroxy-1,4-naphthoquinone |  | 4.11 |  |
| 2-Methyl-5-hydroxy-1,4-naphthoquinone |  | 1.32 |  |
| 2,3-Dichloro-5,8-dihydroxy-1,4-naphthoquinone |  | 1.53 |  |
| 7-Methyl  juglone ethyl acetate |  | 0.22 |  |
| 5-(Benzyloxy)-7-methylnaphthalene-1,4-dione |  | 0.16 |  |
| Propionyl Juglone |  | 0.13 |  |
| 191 |  | 66 | [232] |
| 379 |  | 0.63 |  |
| 415 |  | 5.0 |  |
| Aloesin |  | 38.9 | [233] |
| Aloeresin D |  | 125.3 |  |

**Table S7.** The inhibition potency of other plant-derived compounds against SARS-CoV-2 3CL^pro^**.**

| **Compd.** | **Structure** | **SARS-CoV-2 3CL^pro^**  **IC_50_ (μM)** | **Ref.** |
| --- | --- | --- | --- |
| 1-(Benzo[b]thiophen-2-ylmethyl)  -2,3-dioxoindoline-5-carboxamide |  | 0.053 | [235] |
| 1-((6-Bromonaphthalen-2-yl)methyl)-2,3-dioxoindoline-5-carboxamide |  | 0.047 |  |
| Theaflavin |  | 8.44 µg/mL | [185] |
| Forsythoside E |  | 6.68 | [181] |
| Forsythoside H |  | 10.17 |  |
| Forsythoside I |  | 5.47 |  |
| Isoforsythiaside |  | 5.85 |  |
| Resveratrol |  | 29.81 | [241] |
| All-trans retinoic acid |  | 24.7 | [242] |
| A4 |  | 9.06 | [243] |
| A5 |  | 6.44 |  |
| C5 |  | 4.71 |  |
